# Web-based cardiac **RE**habilitatio**N** alternative for those declining or dropping out of conventional rehabilitation: results of the WREN feasibility randomised controlled trial

**DOI:** 10.1136/openhrt-2018-000860

**Published:** 2018-10-08

**Authors:** Linzy Houchen-Wolloff, Nikki Gardiner, Reena Devi, Noelle Robertson, Kate Jolly, Tom Marshall, Gill Furze, Patrick Doherty, Ala Szczepura, John Powell, Sally Singh

**Affiliations:** 1 Centre for Exercise and Rehabilitation Science (CERS), NIHR Leicester Biomedical Research Centre (BRC)- Respiratory, University Hospitals of Leicester NHS Trust, Leicester, UK; 2 Faculty of Medicine and Health Sciences, University of Nottingham, Nottingham, UK; 3 Department of Neuroscience, Psychology and Behaviour, University of Leicester, Leicester, UK; 4 Institute of Applied Health Research, University of Birmingham, Birmingham, UK; 5 Faculty of Health and Life Sciences, Coventry University, Coventry, UK; 6 Health Sciences, University of York, York, UK; 7 Centre for Technology Enabled Health Research, Coventry University, Coventry, UK; 8 Medical Sciences Division, University of Oxford, Oxford, UK; 9 Department of Infection, Immunity and Inflammation, University of Leicester, Leicester, UK

**Keywords:** cardiac rehabilitation, delivery of care, education

## Abstract

**Introduction:**

Cardiac rehabilitation (CR) is typically delivered in hospital-based classes and is recommended to help people reduce their risk of further cardiac events. However, many eligible people are not completing the programme. This study aimed to assess the feasibility of delivering a web-based CR intervention for those who decline/drop out from usual CR.

**Intervention:**

A web-based CR programme for 6 months, facilitated with remote support.

**Methods:**

Two-centre, randomised controlled feasibility trial. Patients were randomly allocated to web-based CR/usual care for 6 months. Data were collected to inform the design of a larger study: recruitment rates, quality of life (MacNew), exercise capacity (incremental shuttle walk test) and mood (Hospital Anxiety and Depression Scale). Feasibility of health utility collection was also evaluated.

**Results:**

60 patients were randomised (90% male, mean age 62±9 years, 26% of those eligible). 82% completed all three assessment visits. 78% of the web group completed the programme. Quality of life improved in the web group by a clinically meaningful amount (0.5±1.1 units vs 0.2±0.7 units: control). Exercise capacity improved in both groups but mood did not change in either group. It was feasible to collect health utility data.

**Conclusions:**

It was feasible to recruit and retention to the end of the study was good. The web group reported important improvements in quality of life. This intervention has the opportunity to increase access to CR for patients who would otherwise not attend. Promising outcomes and recruitment suggest feasibility for a full-scale trial.

**Trial registration number:**

10726798.

**Key questions**

**What is already known about this subject?**

- Cardiac rehabilitation (CR) is effective in improving patient outcomes and reducing the risk of future cardiac events.
- Despite being recommended in national guidelines, approximately 50% of eligible patients do not take part or do not complete a programme.

**What does this study add?**

- The web-based programme offers an alternative way to complete CR in those who decline or drop out of a conventional hospital programme.
- The wed-based alternative was feasible to deliver and promising signals were noted in secondary outcomes.

**How might this impact on clinical practice?**

- We believe that this web-based approach could provide an acceptable way to increase the provision of CR for those unable (or unwilling) to attend conventional programmes; or to be an alternative mode of CR delivery in a full, choice-based CR menu.
- In turn, this could reduce the risk of future cardiac events in those able to attend a programme.

## Introduction

Coronary heart disease (CHD) affects over 2.3 million people in the UK.^1^ There is a very large cost to both the individual, in terms of loss of quality of life, and to society, in terms of healthcare costs and loss of productivity. It has been estimated that the cumulative cost of cardiovascular disease (CVD) to the UK economy is in the region of £30 billion annually,^2^ through direct service provision, lost productivity and informal care.^3^ The acute and ongoing management of individuals with CHD has been outlined in many national and international guidelines,^4^ and acknowledges the importance of cardiac rehabilitation (CR) in the care pathway of these individuals. CR is commonly a structured and supervised package of care that supports individuals with CHD to achieve their best possible levels of physical, psychological and social function.^5–7^

Many national and international guidelines on management of CHD acknowledge the importance of CR in the care pathway, including the National Institute for Health and Care Excellence (NICE) clinical guideline 172 on myocardial infarction (MI),^8^ 94 on unstable angina and non-ST segment elevation MI^9^ and guideline 108 on chronic heart failure.^10^ The CVD outcomes strategy (2013) set an ambitious target for 65% of people discharged from hospital with a primary diagnosis of acute MI or a surgical revascularisation to be offered CR.^11^

Despite national guidance, the most recent National Audit of Cardiac Rehabilitation indicates that only 51% of eligible patients actually receive CR.^12^ There also appears to be unacceptable variation in uptake (30%–90%) across the UK, underpinned by complex reasons; some related to the organisation and system of delivery^13^ and others to patients’ individual choice. Factors related to rehabilitation non-attendance that have been identified comprised employment commitments, difficulties with transport, lack of time, distance to travel to rehabilitation and embarrassment related to attending rehabilitation.^14–16^ There appears to be some consensus around barriers that argue for the development of alternative formats and modes of rehabilitation delivery, so that access is broadened.^17^ Currently within practice, the ‘Heart Manual’^18^ and the ‘Angina Plan’^19^ are alternative paper-based home rehabilitation options, but are not widely delivered. Studies have shown that most patients with CHD who are still working would prefer a home-based CR programme.^20^ Interestingly, CR delivered either as a supervised or facilitated self-delivered programme has equivalent positive outcomes.^21^ The audit also identified that 33% of patients do not complete a CR programme and the most cited reason for attrition or failure to complete group-based CR is the need to have ‘return to work’.^12^

There is considerable interest in digital health as a means of delivering healthcare for individuals with long-term conditions, where a standard intervention is delivered in a way that is not geographically or time constrained. An increasing proportion of retired people are using the internet, reflecting the typical rehabilitation population. In the UK, 79% and 76% of men and women aged between 65 and 74 years respectively had used the internet within the previous 3 months.^22^ Among adults aged 75+ years, internet use increased from 19.9% to 40.5% from 2011 to 2017 (ie, 3% a year).^22^

Studies carried out across Europe and North America have investigated the efficacy of web-based interventions for those with heart disease.^23–26^ The largest was reported in 2012^26^; however, it was not a comprehensive rehabilitation programme (as defined by the Department of Health’s commissioning pack^27^ and recruited participants with a broad range of CVDs). Reid *et al* 24 reported on a Canadian study which recruited exclusively people postprimary percutaneous coronary revascularisation (percutaneous coronary intervention (PCI) or angioplasty) who were offered a physical activity intervention that was web based. The paper reported a benefit in the intervention arm not observed in the control arm, suggesting the potential value of web-based interventions in this population. The Cochrane review of internet-based interventions for the secondary prevention of CHD, published in 2015, suggested that there was some evidence to support improvements in health-related quality of life and behaviour change, but there was insufficient evidence to draw firm conclusions.^28^

The University Hospitals of Leicester (UHL) NHS Trust has developed a web-based CR programme (‘ACTIVATE YOUR HEART’, www.activateyourheart.org.uk). This online programme was developed over a number of years, and has been tested in two small studies. The first was a pilot randomised controlled trial (RCT) in patients with angina managed exclusively in primary care.^29^ Encouragingly, the data demonstrated improvements in angina symptoms, objectively measured physical activity and levels of anxiety and depression, compared with the control group. A second single cohort observational study collected pilot data from patients attending CR at UHL, which identified significant improvements (p<0.05) in depression, exercise capacity and quality of life (n=106). Interestingly 65% of patients reported that they would not otherwise have attended CR.^30^ This has formed the basis of a case study on the NICE website (http://www.nice.org.uk/usingguidance/sharedlearningimplementingniceguidance/examplesofimplementation/eximpresults.jsp?o=718), encouraging the use and exploration of alternative forms of delivery.

The use of the internet permits greater flexibility of CR delivery, as patients are able to complete their programme at a place and time that suit them. It is also capable of reaching a wider population, especially those patients who live in rural areas.^31^ Studies have highlighted how web-based interventions can also help improve knowledge for patients with chronic health conditions.^32^ There may also be benefits to the service, releasing capacity for CR specialists to manage more complex patients in conventional hospital classes, as well as providing additional choice for those unwilling to do standard CR.^33^

## Objectives

The study aimed to assess the feasibility of delivering a trial offering an alternative web-based CR intervention for those who decline or drop out from conventional supervised CR. The study was a two-centre feasibility study, collecting quantitative data to inform the design of a definitive clinical trial. The specific objectives were to:

1. Derive an estimate of the number of eligible patients at participating centres.
2. Assess the willingness of patients to be randomised to this study and a future trial.
3. Determine opportunities and methods to recruit patients to a future trial.
4. Determine participant adherence to the web-based rehabilitation programme.
5. Test methods for the collection of baseline and follow-up clinical data as well as data completeness and accuracy.
6. Assess the willingness of participants to allow researchers to follow their hospital records/health service data.
7. Identify methods to measure economic costs (health and social care resource use and patientborne costs) and outcomes (including health utility and return to normal work or other activity).

We also collected qualitative data in the form of staff and patient interviews (this will be reported in a separate paper).

## Methods

### Design

A feasibility study to inform the design of a definitive RCT. Patients were randomised to either best usual care or the web-based programme (‘ACTIVATE YOUR HEART’) from two CR centres in the UK (UHL NHS Trust (acute) and Lincolnshire Community Health Services NHS Trust (community)). The trial is registered on the ISRCTN website (ref: 10726798). We aimed to recruit people who declined, or were unable to take up or dropped out of conventional rehabilitation. These participants were recruited from different stages of the rehabilitation pathway, including those identified from the CR database, or those declining the offer of rehabilitation at the time of the initial assessment prior to enrolment onto a conventional programme. Outcome measures were collected at baseline, 8 weeks and 6 months.

### Participants

#### Inclusion criteria

- Confirmed primary diagnosis of CHD (including angina, post-MI, post-PCI).
- Eligible for conventional CR (eligibility as described in guidance from the British Association for Cardiovascular Prevention and Rehabilitation—updated 2017).^34^
- Access to, familiarity with and ability to use the internet. Questions were asked to establish familiarity with the internet, for example, use of either online shopping/online banking.
- People who have recently (<12 months) declined, or were unable to take up the invitation of rehabilitation. These people are defined as those expressing an unwillingness to attend any further stages of the programme either at patient assessment or when the patient care plan was developed.
- People who have recently (<12 months) ‘dropped out’ of rehabilitation. These people are defined as those not attending two consecutive sessions of the comprehensive rehabilitation programme.

#### Exclusion criteria

- No access to the internet, unfamiliar with or unable to use the internet.
- Individuals who have completed rehabilitation for a previous admission in the last 12 months.
- Those demonstrating high levels of depression (defined by baseline Hospital Anxiety and Depression Scale (HADS) score^35^ >11 (moderate depression)) and poor exercise capacity (defined by poor performance on the incremental shuttle walking test (ISWT),^36^ level achieved <3—equivalent to walking 120 m).
- Unable to read English (as the intervention is currently only available in English).

#### Randomisation

Participants were randomised into one of two groups: the intervention group (web-based CR programme) and the control group (usual care) using a web-based randomisation system (www.sealedenvelope.com). Randomisation was stratified by centre, 30 patients by site, 60 in total. Randomisation was performed using permuted blocks, with 60% to the intervention and 40% to the control group. Unequal randomisation was performed to allow for more experience with the intervention group and more data (power) in this group to detect adverse events.

### Trial interventions

#### Intervention group: web-based CR programme

‘ACTIVATE YOUR HEART’ (www.activateyourheart.org.uk) is an online intervention designed for participants to use self-directed at home, facilitated with remote support from the CR team. The broad aim of the programme is to improve the overall cardiac risk factor profile of patients. The intervention is an interactive, password-protected, tailored CR programme, contained in a website. The programme was developed at the UHL NHS Trust and coproduced with healthcare professionals, a software development team (HARK2) and a group of patients/members of the public.

The programme contains four stages and can be completed in 8 weeks but access to the site and its features continues for 12 months. Before beginning the programme each patient receives face-to-face training on the website and a written user manual. They then complete an online registration form, providing information about their current and previous medical history and their cardiac risk factors. The website uses this information as baseline measurement, and to create an individually tailored plan for the patient. Throughout the programme, patients have access to a discussion forum and an ‘ask the expert’ email facility. CR staff at both centres had access to the administration side of the website; here they are able to monitor each participant’s progress. Participants and the CR team were alerted whenever the programme was not being used regularly. More details about the programme are reported in a previous paper.^30^

#### Control group

Participants in this group received best usual care for their region. Usual care in the absence of CR would comprise a referral back to general practice and general advice in the form of standard verbal advice and guidance booklets. All participants in the control group were given the opportunity to participate in the ‘ACTIVATE YOUR HEART’ programme following their 6-month follow-up appointment.

#### Outcome measures

The primary outcome measure in this study was to assess the feasibility of recruiting/retaining people who met the inclusion criteria; those who have declined/dropped out of traditional CR.

We also examined the:

- Safety of the trial (adverse event reporting and angina symptom diary).
- Feasibility of our randomisation process and willingness to be randomised.
- Retention rate of participants to the study at 8 weeks and 6 months of follow-up.
- Feasibility of conducting the outcome measures which are proposed for a definitive trial, collected at blinded 8-week and 6-month assessments including:
  - Health-related quality of life: the MacNew Heart Disease Questionnaire,^37^measuring physical, emotional and social aspects of quality of life.
  - Exercise capacity: measured using the ISWT,^36^ which is a test used to assess cardiorespiratory fitness. An initial practice test was conducted at baseline to minimise any possible learning effect.
  - Anxiety and depression: measured using the HADS.^35^
  - Self-efficacy: measured using ‘the general self-efficacy scale.^38^
  - Resource use (health/social care and personal costs) questionnaire for healthcare and other services received: to identify methods to measure costs (health and social care resource use and patient borne costs) and outcomes (including health utility and return to normal work or other activity). The questionnaire was devised for the purposes of this study by one of the authors (AS) and can be found here: http://www.dirum.org/instruments/details/104.

### Non-clinical study outcomes/process measures

- Web usage: total web usage statistics for patients assigned to the web-based programme were monitored, along with emails sent to the expert CR team.
- Intervention completion rates among intervention group participants.

#### Sample size estimation and recruitment target

As this was a feasibility study, a formal sample size calculation was not required to detect between-group changes. We therefore aimed to recruit 30 individuals at each site within the recruitment phase of the study, 60 individuals in total. This was a conservative number, anticipating that each site will recruit approximately three participants per month. Data from 60 participants were deemed reasonable to assess the recruitment/retention rate and allow for planning the subsequent final trial by obtaining estimates of potential outcomes (ie, health-related quality of life) with sufficient precision (ie, with an SE of less than 8% for estimated proportions). This is in keeping with recommendations of 30 participants required in feasibility/pilot studies to estimate a parameter.^39^ Furthermore, a recent audit of UK feasibility studies found that the median sample size for a two-arm trial was 36 and 30 per arm, respectively, for dichotomous and continuous endpoints.^40^

#### Quantitative data analysis

Data were entered and stored on a secure web-based system (REDCAP) which has discrepancy management features. Data were then transferred from REDCAP to the Statistical Package for the Social Sciences (SPSS) V.18 (SPSS). Analysis was primarily descriptive, that is, estimation of means and SDs, proportion of patients eligible/willing to participate in the study, dropouts and completion rates. In line with the accepted practice for feasibility studies, no p (significance) values/inferential statistics are presented.

## Results

**Figure 1:**
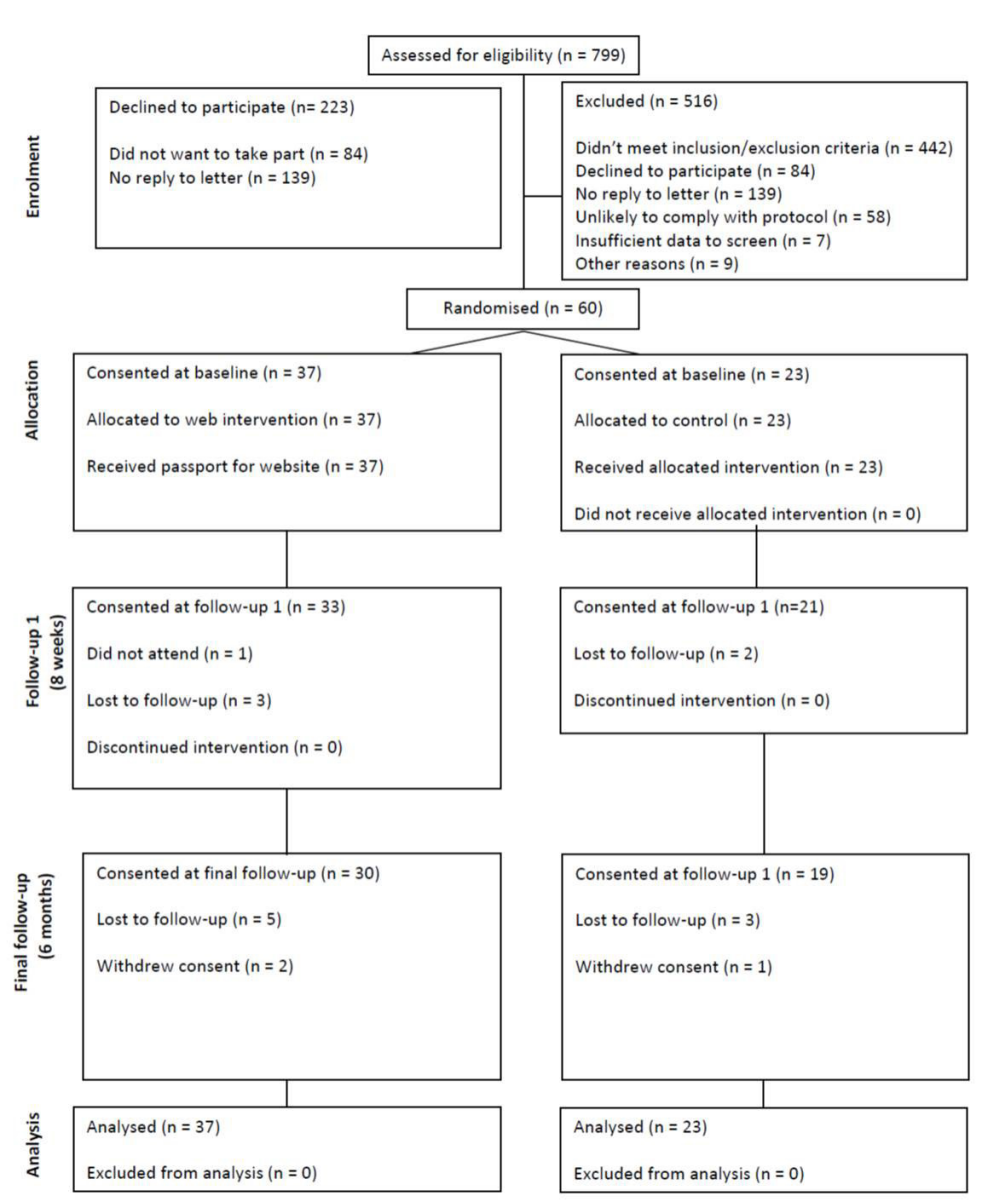
Consolidated Standards of Reporting Trials (CONSORT) diagram by treatment group.

Sixty patients were recruited and randomised to the study between December 2015 and April 2017: 37 to the web group and 23 to the control group. Figure 1 shows the flow of eligibility, screening, randomisation and follow-up in the study. The main reason for exclusion to the study was that patients did not meet the inclusion criteria, n=442 (ie, no confirmed diagnosis of CHD, completed CR in the last 12 months, comorbidities/contraindications to exercise or not web literate). Of those eligible to be randomised, n=60 or 26% were willing to take part in the trial. Tables 1 and 2 show the baseline characteristics and clinical outcomes of the two groups. The groups appear well matched at baseline though no statistical tests were performed to confirm this.

**Table 1:** Baseline characteristics of the two groups

	Web group(n=37)	Control group(n=23)
Gender		
Male, n (%)	33 (89)	21 (9)
Age	
Mean (SD)	62 (10)	61 (8)
Ethnicity		
White, n (%)	37 (100)	21 (91)
Marital status		
Married, n (%)	27 (75)	17 (74)
BMI
Mean (SD)	28 (11)	29 (12)
Family history
Yes, n (%)	20 (54)	6 (26)
Employment status, n (%)		
Employed	14 (38)	9 (39)
Self-employed	1 (3)	1 (4)
Retired	21 (57)	12 (52)
Part-time and retired	1 (3)	0 (0)
Unpaid work and retired	0 (0)	1 (4)
Previous cardiac event, n (%)		
IHD (Ischaemic Heart Disease)	7 (19)	1 (4)
Angina	9 (24)	6 (26)
PCI	6 (16)	4 (17)
Cardiac arrest	5 (14)	2 (9)
Other	10 (27)	10 (44)
Medication, n (%)		
Beta blockers	33 (89)	18 (78)
ACE (Angiotensin Converting Enzyme) inhibitors	32 (87)	19 (83)
GTN (Glyceryl Trinitrate) spray	29 (78)	20 (87)
Anticoagulants	19 (51)	11 (48)
Aspirin	35 (95)	21 (91)
Statins	35 (95)	21 (91)
Diuretics	9 (24)	2 (9)
Calcium channel blocker	4 (11)	5 (22)
Antiarrhythmic	6 (16)	16 (70)

BMI, body mass index;GTN, Glyceryl trinitrate; IHD, Ischeamic heart disease; PCI, percutaneous coronary intervention.

**Table 2:** Clinical outcome measures at baseline, 8-weeks and 6-months in both groups. Results are presented as mean (SD)

	Baseline		8 weeks		6 months	
	Web	Con	Web	Con	Web	Con
MacNew overall score	5.9 (1.0)	6.0 (0.8)	6.2 (0.7)	6.2 (0.6)	6.5 (0.5)	6.2 (0.6)
ISWT (m)	437.6 (153.6)	458.0 (153.6)	479.0 (180.1)	492.0 (167.9)	491.3 (192)	564.7 (152.9)
HADS Anxiety Score	3.9 (3.2)	4.0 (2.7)	3.4 (3.6)	4.8 (3.6)	3.0 (3.3)	3.8 (3.0)
HADS Depression Score	2.7 (2.7)	2.5 (2.6)	1.7 (2.1)	2.4(2.2)	1.5 (1.4)	2.4 (2.5)
General Self-Efficacy Scale score	33.6 (4.3)	35.4 (4.0)	35.2 (3.5)	35.3 (4.8)	35.8 (3.6)	35.7 (4.2)

Results are presented as mean (SD).

HADS, Hospital Anxiety and Depression Scale;ISWT, incremental shuttle walking test.

More patients dropped out/were lost to follow-up from the web intervention group (n=7) compared with four in the control group by 6 months.

Retention rates in the trial were excellent: overall 54 patients attended the 8-week assessment (90%; 95% CI 79% to 96%) and 49 patients attended the 6-month assessment (82%; 95% CI 70% to 90%).

There were two adverse events in the web group and four in the control group—but all were deemed unrelated to the study procedures and interventions. At 8 weeks, four patients in the web group and one control patient reported at least one episode of angina in their symptom diaries. These episodes were relieved with glyceryl trinitrate spray and/or rest.

The most fruitful method of recruitment was to capture patients at the point of declining rehabilitation in a one-to-one assessment (>80% of those recruited) compared with retrospectively contacting those who had declined or dropped out of a programme previously.

table 3 shows the change within groups for the clinical outcomes at 8 weeks and 6 months. The change in the MacNew total score at 6 months met suggested the minimum clinical important difference of 0.5^41^ for this outcome in the web group.

**Table 3:** Changes in clinical outcome measures at 8 weeks and 6 months in both groups

	8 weeks		6 months	
	Web	Control	Web	Control
MacNew overall score	0.3 (1.0)	0.2 (0.8)	0.5 (1.1)	0.2 (0.7)
ISWT (m)	45.5 (57.0)	50.0 (76.9)	52.9 (76.8)	85.9 (115.2)
HADS Anxiety Score	−0.3 (3.0)	0.7 (3.1)	−0.4 (3.3)	−0.2 (2.8)
HADS Depression Score	−0.3 (3.0)	0.7 (3.1)	−0.8 (2.6)	0.1 (1.6)
General Self-Efficacy Scale score	1.5 (4.1)	−0.1 (2.9)	2.1 (5.1)	−0.3 (1.6)

Results are presented as mean (SD).

HADS, Hospital Anxiety and Depression Scale;ISWT, incremental shuttle walking test.

It was feasible to measure costs (health and social care resource use and patientborne costs) and outcomes (including health utility and return to normal work or other activity) using the Resource Use Questionnaire designed for this study—see here: http://www.dirum.org/instruments/details/104. The overall completion of this questionnaire was 90% across both groups at 6 months. In future work, both costs and outcomes will be analysed and reported using standard national Health Technology Assessment Framework standards.^42^

**Figure 2:**
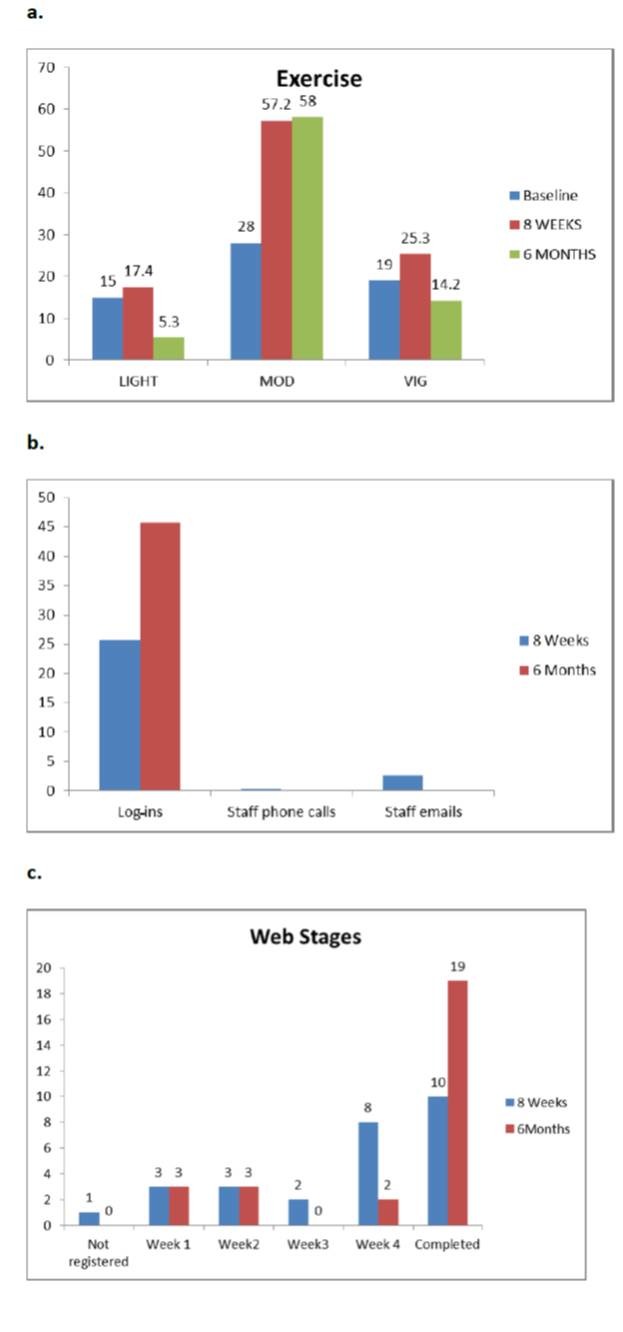
Web usage statistics. (A) Average minutes of exercise logged per week. (B) Average number of log-ins, phone calls and emails. (C) Completion of web stages.

Web usage statistics are shown in figure 2A–C. Patients were able to double their exercise time (in minutes) from baseline to 8 weeks and this was maintained at the 6-month assessment. The average number of log-ins was three times per week at the 8-week mark and twice a week at 6 months. Phone calls and emails from patients to staff were low and patients did not use the group forum. Twenty-nine (of the 37 participants) had completed the web programme at 6 months (78%) and some were still working their way through the programme.

## Discussion

CR is a highly effective intervention in the management of patients with CHD.^5^ Despite its numerous benefits, it is an underused treatment, with around only half of eligible patients in the UK and around 30% in Europe accessing a programme.^12 43^ Alternative or more flexible ways of delivering CR therefore ought to be considered.

The data from this study show that a web-based CR programme (‘ACTIVATE YOUR HEART’) has the potential to be an acceptable way to increase the provision of CR for those unable (or unwilling) to attend a conventional programme. If alternative forms of CR allow more patients to access a service, this in turn could reduce the risk of future cardiac events for those able to attend a programme.

Of those who were eligible, a quarter agreed to participate. For future studies, we may need to review the inclusion criteria to allow those with more complex needs to access the web programme (including those with low shuttle walk test performance or high levels of anxiety or depression). Also, we expect that over time, the numbers of patients who are web literate will increase. This would be in line with the recent internet use statistics revealing that internet use in those aged 65 years and over is catching up with the younger age groups.^22^ Furthermore, we are aware that the NHS priorities around the use of technology in healthcare are changing and we would expect the capacity for digital health interventions to continue to grow. The NHS has pledged in its 5-year plan to ‘train our staff so that they are able to support those who are unable or unwilling to use new technologies.’^44^

We were encouraged by the high levels of engagement with the web programme, as measured by numbers of log-ins, exercise sessions logged and that 78% had completed the programme at 6 months (with others still working their way through the programme). It was perhaps surprising that patients took longer than the anticipated 8 weeks to complete the online programme. However, patients had access to the ‘ACTIVATE YOUR HEART’ programme for 1 year, therefore the programme can be completed at the patients’ own pace. Furthermore, access to the programme for this protracted period has the potential to improve maintenance, though we did not measure long-term follow-up of outcomes as part of this study. Interestingly, patients required minimal support from healthcare staff in terms of phone calls and emails. As this is the only part of web-based CR programme where costs increase with increasing numbers of participants (increased staff time); this suggests that the costs with increasing numbers will remain low. We were able to identify the best method of recruitment in this feasibility study. This was to approach patients prospectively at the point of declining rehabilitation in a one-to-one assessment (>80% of those recruited) as opposed to retrospectively contacting those who had declined or dropped out of a programme previously. It may be that uptake at this stage was also influenced by the healthcare professional introducing the research study (ie, the clinical rehabilitation team and not the research team). We would use this recruitment strategy going forward to a full trial. However, we should not limit the offer of web-based rehabilitation to only those who decline a conventional, class-based programme. Last year, 65 344 eligible patients missed out on a life-saving intervention that also improves quality of life.^12^ CR ought to be a full menu with genuine choice and resources that support patient preference. Web-based rehabilitation is one of the innovations required to future proof CR.

Furthermore, it was feasible to collect all clinical, non-clinical and health utility outcomes in this trial. Finally, there was excellent completion of all study outcomes and procedures, with 82% of individuals attending all three assessment visits.

While this feasibility study was not powered to test clinical effectiveness of the web programme, we are encouraged by the clinically worthwhile gains shown in the MacNew Quality of Life Questionnaire in the web group and some small positive signals in the other outcome measures. Overall, we were able to show that the web-based CR programme was safe (low adverse events and attrition unrelated to the study intervention) and could secure improvements associated with conventional CR. Mood (anxiety and depression) and self-efficacy did not really improve in either group, though it is important to note a potential floor effect since the groups both had low levels of anxiety and depression and high self-efficacy scores at the programme’s start, reducing scope for change. We did not anticipate the large change in the ISWT of the control group at 6 months (86 m vs 53 m in the web group), and are unsure of the reasons for this change. It may be argued that the mere act of performing an outcome measure may influence the subsequent outcome. In other words, the performance of an ISWT in itself could be considered an intervention and have an effect on the patients’ confidence to complete that test.^45 46^ However, as stated previously, the self-efficacy scores (an indicator of confidence) did not improve in either group. In a full-scale trial, it may be appropriate to perform a sensitivity analysis to examine the extent to which results are ‘affected by changes in methods, models, values of unmeasured variables, or assumptions.’^47^

## Conclusion

This feasibility study of web-based CR versus usual care has provided us early signs of patient benefit and produced useful information about how best to recruit to a definitive trial. We have been encouraged by some promising outcomes and the data suggest feasibility for a full-scale trial. There is the potential for a trial looking at the effectiveness of the web-based programme in decliners. There is also the scope to evaluate web-based CR as part of a full menu of options.

This intervention has the opportunity to increase access to CR for patients who would otherwise not attend. Or to be an alternative mode of CR delivery in a full, choice-based CR menu. In turn, this could reduce the risk of future cardiac events and therefore be cost saving.

10.1136/openhrt-2018-000860.supp1 Abstract translation This web only file has been produced by the BMJ Publishing Group from an electronic file supplied by the author(s) and has not been edited for content.
