# Supplementary material for: Web-based cardiac REhabilitatioN alternative for those declining or dropping out of conventional rehabilitation: results of the WREN feasibility randomised controlled trial
Source: Open Heart. 2018 Oct 8;5(2):e000860. doi: 10.1136/openhrt-2018-000860 (PMC6196944; doi:10.1136/openhrt-2018-000860)
Supplement: Abstract translation [file openhrt-2018-000860supp001.docx]

**Abstract**

**Introduction:** Cardiac Rehabilitation (CR) is typically delivered in hospital-based classes and is recommended to help people reduce their risk of further cardiac events. However, many eligible people are not completing the programme. This study aimed to assess the feasibility of delivering a web-based CR intervention for those who decline/ drop-out from usual CR.

**Intervention:** a web-based CR programme for 6-months, facilitated with remote support.

**Methods:** Two-centre, randomised controlled feasibility trial. Patients were randomly allocated to web-based CR/ usual care for 6-months. Data was collected to inform the design of a larger study: recruitment rates, quality of life (MacNew), exercise capacity (incremental shuttle walk test) and mood (Hospital Anxiety and Depression Scale). Feasibility of health utility collection was also evaluated.

**Results:** 60 patients were randomised (90% male, mean age 62 ± 9 years, 26% of those eligible). 82% completed all three assessment visits. 78% of the web group completed the programme. Quality of life improved in the web group by a clinically meaningful amount (0.5 ± 1.1 units Vs 0.2 ± 0.7 units: control). Exercise capacity improved in both groups but mood did not change in either group. It was feasible to collect health utility data.

**Conclusions:** It was feasible to recruit and retention to the end of the study was good. The web group reported important improvements in quality of life. This intervention has the opportunity to increase access to CR for patients who would otherwise not attend. Promising outcomes and recruitment suggest feasibility for a full scale trial.
